# Cognitive Coping Strategies among Inpatient Adolescents with Depression and Psychiatric Comorbidity

**DOI:** 10.3390/children10121870

**Published:** 2023-11-29

**Authors:** Ilinca Mihailescu, Magdalena Efrim-Budisteanu, Lucia Emanuela Andrei, Alexandra Mariana Buică, Mihaela Moise, Ingrid Georgiana Nicolau, Alexandra Diana Iotu, Adriana Petruța Grădilă, Teodora Costea, Agnes Maria Priseceanu, Florina Rad

**Affiliations:** 1 Child and Adolescent Psychiatry Department, Carol Davila University of Medicine and Pharmacy, 020021 Bucharest, Romania; ilinca.mihailescu@umfcd.ro (I.M.); lucia.andrei@drd.umfcd.ro (L.E.A.); alexandra.buica@umfcd.ro (A.M.B.); mihaela.stancu@drd.umfcd.ro (M.M.); florina.rad@umfcd.ro (F.R.); 2 Child and Adolescent Psychiatry Department, “Prof. Dr. Al. Obregia” Clinical Hospital of Psychiatry, 041914 Bucharest, Romania; ingrid-georgiana.nicolau@rez.umfcd.ro (I.G.N.); alexandra-diana.iotu@rez.umfcd.ro (A.D.I.); petruta-adriana.gradila@rez.umfcd.ro (A.P.G.);; 3 Faculty of Medicine, Titu Maiorescu University, 040441 Bucharest, Romania; 4 Psychology Independent Practice, 110174 Pitești, Romania; psihologpriseceanuagnes@gmail.com

**Keywords:** adolescent depression, cognitive strategies, coping, depression, emotional regulation, inpatients

## Abstract

The aim of the present study is to describe and measure the cognitive emotion regulation strategies of inpatient adolescents with clinical depression, aged 13–18, and to analyse these coping strategies in relation to different comorbidities of Major Depressive Disorder (MDD). Methods: There were 112 adolescents with MDD who were admitted to hospital and 78 healthy adolescents included in the study. The Cognitive Emotion Regulation Questionnaire (CERQ) was used to assess nine specific cognitive coping strategies. A cognitive coping style model for depression in adolescents was described by analysing the differences between the two groups. The CERQ scores in MDD participants, grouped by comorbidity, were also assessed. Results: Adolescents with MDD had significantly higher scores for Self-Blame and Catastrophising strategies, and significantly lower scores for Positive Refocusing, Refocusing on Planning, and Positive Reappraisal. Adolescents with MDD and Borderline Personality Disorder (BPD) traits had significantly higher scores for Rumination, Catastrophising, and Blaming Others than adolescents with MDD and anxiety or with no comorbidity. Conclusions: Clinical depression in adolescents is associated with a cognitive profile that consists of an increased use of maladaptive coping styles and low employment of adaptive strategies. Early identification can contribute to the development of specific, individualised prevention and intervention programmes, while further longitudinal studies are necessary to adequately measure the outcome of these interventions.

## 1. Introduction

Major Depressive Disorder (MDD) constitutes a global public health issue and a major burden of disease for adolescents, with a point prevalence of 8% and a lifetime prevalence of 19%, while 34% of adolescents worldwide are at risk of developing MDD [1]. Depression in children and adolescents is frequently associated with other mental health disorders, which influences the prognosis, treatment, quality of life, and overall burden of the disease. It is estimated that 28% of children with depression have a comorbid anxiety disorder, 7% have Attention Deficit Hyperactivity Disorder (ADHD), and 6% have conduct and oppositional defiant disorder [2]. Moreover, individuals with Autism Spectrum Disorder (ASD) have a four-times-higher chance of developing depression when compared to typically developed peers [3], and the reported point prevalence of MDD among youths with ASDsvaries between 0 and 88% [4]. Another clinical aspect that can complicate the presentation, making the diagnosis, treatment, and management even more difficult, is the presence of personality traits along with depression. Although there is no consensus whether Borderline Personality Disorder (BPD) represents a well-founded diagnosis in adolescence, studies estimate that up to 5% of adolescents meet the Diagnostic and Statistical Manual of Mental Disorders, Fifth Edition (DSM 5) criteria for BPD, and that the presentation is more severe in adolescents compared to adults [5].

Although cognitive emotion regulation strategies are difficult to define and measure, they are typically described as mental “responses to emotion-eliciting events that consciously or unconsciously attempt to modify the magnitude and/or type of individuals’ emotional experience or the event itself” [6,7]. These responses can be classified as either adaptive or maladaptive, depending on their impact on the mental state and their relation to psychopathology [8]. Garnefski et al. describe Positive Refocusing, Positive Reappraisal, Refocusing on Planning, Putting into Perspective, and Acceptance as adaptive strategies, while Rumination, Catastrophising, Self-Blame, and Blaming Others are considered to be maladaptive [8,9].

To understand emotion regulation strategies in children and adolescents and their effects, a developmental perspective should be taken into consideration, because of the different levels of biological, cognitive, and emotional maturation [10]. The way in which children and adolescents manage to cope with stressful situations and to regulate their emotions in response to different, difficult events could model future emotional, cognitive, and social development, but, conversely, a child’s current development may shape their coping strategies [10]. Moreover, the pattern of coping mechanisms used by a developing child impacts their future resilience, and it may, therefore, decrease or increase the chances of a child developing a mental health disorder [11]. Previous studies analysing the cognitive regulation of emotions and its correlation with psychopathology have more frequently addressed adult populations rather than children/adolescents, and general community populations rather than clinical ones. In general, the use of maladaptive strategies, such as, but not limited to, Catastrophising, Rumination, and Suppression, has been correlated with mental health disorders, such as depression [9,12,13], anxiety disorders [14,15], PTSD [16,17], eating disorders [18,19], etc.

Regarding the relationship between different coping strategies and depression in adolescents, a study carried out on a non-clinical adolescent sample identified a specific cognitive model for depression in adolescents, which was also different from the one for anxiety [15]. High Rumination and Self-Blame (only in females), and low Positive Refocusing and Positive Reappraisal (in both males and females), were significantly associated with depression [15]. A meta-analytic review of Schäfer et al. in 2016, considering the relationship between psychopathology and six cognitive strategies (i.e., Problem Solving, Cognitive Reappraisal, Acceptance, Rumination, Suppression, and Avoidance), included 68 effect sizes from 35 studies and showed that all six strategies were significantly related to depression symptoms in adolescents [20]. In this meta-analysis, there were negative associations with depression, with medium effect sizes for Problem Solving, Cognitive Reappraisal, and Acceptance; and positive associations, with large effect sizes for Rumination and Avoidance and only small effect sizes for Suppression [20]. It is worth mentioning that all the above-mentioned studies included in the meta-analysis were conducted on either school or community samples.

The aim of the present study is to describe and measure the cognitive emotion regulation strategies of inpatient adolescents with clinical depression, aged 13–18, and to analyse coping strategies across adolescents with different comorbidities of MDD. We hypothesised that a profile would emerge, with adolescents with MDD showing a higher level of maladaptive strategies and a lower level of adaptive ones compared to healthy adolescents. It was also hypothesised that the presence of different comorbidities with MDD would impact this profile, with different cognitive strategies being used by adolescents diagnosed with anxiety, ASD, or BPD traits.

## 2. Materials and Methods

### 2.1. Participants and Data Collection Procedure

One hundred and ninety adolescents (N = 190, 144 female and 46 male) between 13 and 18 years of age (M = 15.06, SD = 1.70) participated in this study. The study group consisted of 112 adolescents (90 female and 22 male) with MDD, aged 13–17 (M = 14.91, SD = 1.55). The inclusion criteria for the MDD group were adolescents aged between 13 and 18 years, who met the DSM 5 criteria for MDD, and who gave their assent to participate in the study. The exclusion criteria for the MDD group were those with current or past psychotic disorders and intellectual disability (IQ < 70). The control group comprised 78 healthy adolescents (54 female and 24 male), aged 13–18 (M = 15.28, SD = 1.89). The eligibility criteria for the control group were adolescents aged between 13 and 18 years, who had no current or past psychiatric diagnosis, and who gave their assent to participate in the study.

Data were collected during a 1-year time frame, between May 2022 and May 2023. For the MDD group, cases were identified upon admission to the “Prof. Dr. Alexandru Obregia” Clinical Hospital of Psychiatry through psychiatric evaluation, and then referred to the research team. A signed consent form was obtained from the parents of the referred adolescents, and a second psychiatric evaluation was performed in order to assess their eligibility for participation in the study. Participants included in the study completed the Cognitive Emotion Regulation Questionnaire (CERQ) under the supervision of one member of the research team. The participants in the study group were therefore all the patients admitted to an inpatient psychiatric hospital who received a clinical diagnosis of MDD and met the inclusion and exclusion criteria during a 1-year time frame. The healthy control group was recruited from community high schools in the area and from a convenience sample of the hospital staff’s children. Adolescents in the control group completed an evaluation conducted by one of the members of the research team to assess the eligibility criteria, and a written consent form was obtained from the parents. The study received approval from the Research Ethics Board of the “Prof. Dr. Alexandru Obregia” Clinical Hospital of Psychiatry (24330, 7 July 2022). 

### 2.2. Measures

The Cognitive Emotion Regulation Questionnaire (CERQ) [9,21]) was used to assess specific cognitive coping strategies. The CERQ is a 36-item multidimensional questionnaire that identifies nine different types of cognitive coping strategies: Self-Blame, Acceptance, Rumination, Positive Refocusing, Refocusing on Planning, Positive Reappraisal, Putting into Perspective, Catastrophising, and Blaming Others. Each participant rates the items on a five-point Likert scale: “(almost) never” (1), “sometimes” (2), “usually” (3) “often” (4) and “(almost) always” (5), depending on how often they use certain cognitive coping strategies. Higher raw scores on a subscale indicate that the strategy is used more often by the participant. The psychometric properties of the Romanian version of the CERQ have been reported in a previous study conducted on a non-clinical population of 368 Romanian adolescents [21]. The internal consistency estimates were considered to be acceptable for the majority of the subscales (α = 0.63 to α = 0.79), with the exception of Acceptance, for which they were slightly lower (α = 0.59) [21]. The test–retest reliability coefficients for all the subscales were adequate to good, with values ranging from 0.48 to 0.65 [21]. Correlations between the CERQ subscales were examined to determine to what extent the scales are interrelated, with most of the Pearson coefficients being below <0.50. Several high correlations (r ≥ 0.50) were found between Positive Reappraisal and either Refocusing on Planning or Putting into Perspective [21]. 

### 2.3. Data Analysis

Statistical analysis was performed using SPSS version 17.0 (SPSS Inc. Released 2008. SPSS Statistics for Windows, Version 17.0. SPSS Inc., Chicago, IL, USA). Descriptive statistics was used to determine the mean, standard deviation, median, frequency, and percentage for the demographic, clinical, and CERQ data. Non-parametric tests were used to analyse group differences in the CERQ scores. Spearman’s rho was used to determine the correlations between different CERQ strategies. One-way ANOVA on ranks with post-hoc analyses were conducted to compare the CERQ scores in MDD participants, grouped by comorbidity. Statistical significance was set at *p* < 0.05. Effect sizes were also reported (η^2^) to determine the magnitude of the obtained differences. An a priori power analysis was performed using G*Power3 [22] to examine the difference between the means of two independent groups on the CERQ scales, with a medium effect size (d = 0.50) and an alpha of 0.05. The results showed that a total sample of 140 was required to achieve a power of 0.80.

## 3. Results

Table 1 displays the mean, standard deviation, skewness, kurtosis of the CERQ scales in the MDD and Control groups, as well as Cronbach’s alpha coefficients. The CERQ data were non-normally distributed, as assessed by examining the descriptive statistics and also using normality tests. The results obtained on the CERQ showed significantly higher scores in the MDD group using the Self-Blame and Catastrophising strategies, and significantly lower scores using Positive Refocusing, Refocusing on Planning, and Positive Reappraisal (Table 2). The differences for those using Self-Blame and Positive Refocusing had small effect sizes, while those using Catastrophizing, Refocusing on Planning, and Positive Reappraisal had medium effect sizes (Table 2). No differences between the two groups were found for Rumination, Acceptance, Putting into Perspective, and Blaming Others (Table 2). 

Overall, maladaptive cognitive strategies were reported to be used less often than adaptive strategies in the Control group and more often in the MDD group. Thus, the most frequently used strategies in the Control group were Positive Reappraisal, Refocusing on Planning, and Acceptance, while Self-Blame, Rumination and Acceptance were the strategies most often reported by the participants in the MDD group (see Table 1). 

Cronbach’s alpha coefficients were calculated for all the CERQ scales, in both groups (Table 1). The majority of Cronbach’s alpha coefficients were acceptable or almost acceptable (from nearly 0.70 to 0.86) in both groups, with two exceptions: Acceptance in the MDD group (α = 0.48) and Positive Refocusing in the Control group (α = 0.58). The same scales had acceptable and good reliabilities in the other group, and, given the small number of items per scale, it was decided that no adjustments were needed for the present study. 

We further explored the CERQ data in the MDD group, considering the comorbidities of the participants (ASD, BPD traits, anxiety, and no comorbidity) (Table 3). The results of the one-way ANOVA on ranks (Kruskal–Wallis H test) showed statistically significant differences between the different comorbidities for the Rumination score H(3) = 8.012, *p* = 0.046, the Catastrophising score H(3) = 8.625, *p* = 0.035, and the Blaming Others score H(3) = 13.878, *p* = 0.003. 

We conducted post hoc analyses to test pairwise comparisons of each of the three significant Kruskal–Wallis H test results. The MDD + BPD Group had significantly higher Rumination scores than the MDD + ASD Group, the MDD + anxiety Group, and the MDD and no comorbidity Group, with no other differences between the other groups. The post hoc analysis showed significantly higher Catastrophising scores in the MDD and BPD Group, when compared to the MDD + anxiety Group, with no other differences between the other groups using this strategy. The BPD + MDD Group had significantly higher scores for the Blaming Others strategy than the MDD + anxiety Group and the MDD and no comorbidity Group. The MDD + ASD Group also had higher Blaming Others scores than the MDD + anxiety Group, with no significant differences between the MDD + ASD Group and the MDD + BPD Group. 

## 4. Discussion

### 4.1. Coping Cognitive Strategies in Inpatient Adolescents with Depression

The first aim of this study was to describe the pattern of cognitive emotion regulation strategies in a clinical sample of adolescents with MDD that were admitted to a psychiatric inpatient unit during the study period. The strategies with the highest scores in the MDD group were Self-Blame, Rumination, and Acceptance, while healthy participants in the control group had the highest scores for Refocusing on Planning, Positive Reappraisal, and Acceptance. When comparing the two groups, statistically significant differences were observed: adolescents with MDD were significantly more likely to use Self-Blame and Catastrophising and less likely to use Positive Refocusing, Refocusing on Planning, and Positive Reappraisal. The differences with the largest effect sizes in our study were observed for using Catastrophizing, Refocusing on Planning, and Positive Reappraisal as cognitive coping strategies. 

These results largely overlap with the results of other studies, showing that depression is positively associated with the use of maladaptive coping strategies (e.g., Rumination, Catastrophising) and negatively associated with the use of adaptive strategies (e.g., Positive Reappraisal). 

There are some aspects worth discussing regarding the results of the present study and their implications. Firstly, most of the previous conclusions regarding the relationship between cognitive strategies and psychopathology among adolescents were reached from studies conducted on community samples of children and adolescents. The adolescents in the present study are patients with MDD hospitalised during the course of the study. This implies that they represent severe cases, either according to the severity of the current episode, or according to the suicidal risk, the resistance to treatment, the presence of multiple comorbidities, or any other clinical feature that could have led to the decision to be hospitalised. The fact that the severity of a case does not really modify the cognitive model of the strategies used in stressful situations could have positive implications. It could show the predictability of the coping mechanisms during the evolution of the disorder, which could also affect the intervention programmes that will, as a result, not require significant adaptations to the targeted cognitive strategies. By linking the fact that there is no specificity related to severity with the fact that there is no age-specificity (the cognitive style associated with depression does not undergo significant changes in adults), implications for prevention can result as well [15]. Thus, via the early correction of the formation of some non-adaptive strategies and especially via an increase in the use of adaptive mechanisms, we can prevent the installation of maladaptive lifetime coping styles, possibly preventing adolescent and adult psychopathology.

Another result that should be mentioned is related to the scores obtained for the Acceptance strategy, which were similar in adolescents with depression and in healthy adolescents. There are several possible explanations for this finding, but it is most likely that the result can be related to an ambivalent understanding of acceptance. A review of the topic of acceptance as either an adaptive or a maladaptive coping reaction underlines the fact that the operationalisation of the term is controversial [20]. Thus, there is a possibility that two types of acceptance can be hidden behind this construct: the first one, called active acceptance, involves recognising a situation as being negative, followed by managing it constructively, leading to a positive state of mind and wellbeing; the second one, called resigning acceptance, has negative effects on the emotional state, and leads to the abandonment of proactive actions, a lack of hope, and generally a passive attitude [20]. It is thus possible that the participants from the present study use this strategy in different ways, so that the potential differences between depressed and healthy adolescents cease to be visible. 

### 4.2. Cognitive Strategies across Different Comorbidities

The adolescents with depression included in the study were later divided according to their association with other mental health problems (anxiety disorders, ASD, BPD traits, and without other comorbidities), in order to compare their cognitive coping styles depending on the association with different comorbidities. The main finding is that adolescents with depression and BPD traits generally reported higher scores for the maladaptive strategies and lower scores for the adaptative ones compared to the remainder of the adolescents with depression. Statistically significant differences were observed for the Rumination, Catastrophising, and Blaming Others scores, since adolescents with depression and BPD traits have reported using these strategies even more frequently. The result is not unexpected and is entirely in line with other studies [23,24,25], considering the fact that difficulties in tolerating and regulating intense emotions are part of the main characteristics of BPD, leading to the use of dysfunctional behaviours for emotional regulation. 

Overall, there are no significant differences in the majority of strategies between the adolescents with depression and ASD and the adolescents with depression and no ASD, with only one exception related to the Blaming Others scores, which were similar to the ones observed in the MDD + BPD traits group. A cognitive style that includes Blaming Others as a coping strategy in patients with high-functioning ASD was previously reported by Bruggink et al. in 2016, after controlling for anxiety and depression [25]. The higher Blaming Others scores obtained by adolescents with depression and either BPD traits or ASD can be explained by the use of this maladaptive coping strategy when the stressor comes from social relationships, considering the significant challenges in relationships that patients with both BPD and ASD have. Thus, a study carried out by Garnefski et al. showed that, when the stressful situation comes from relational experiences, adolescents would rather use Blaming Others as a coping strategy, compared to, for example, the use of the Self-Blame strategy, when they are exposed to a health threat experience [26]. Although the current study did not include patients with ASDswithout depression, it seems that the cognitive style observed in our participants with MDD + ASD (apart from Blaming Others) is the one associated with the presence of depression. Additional studies that include patients with ASD without depression are necessary to characterise the emotional regulation modalities specific to ASD. Considering the well-known difficulties related to the theory of the mind, understanding and labelling the emotions of others, and adjusting one’s own emotions, thoughts, and behaviours to external emotional stimuli, it is easy to anticipate that patients with ASD may have difficulties with emotional regulation.

### 4.3. Clinical Implications and Limitations

Several findings from this study can contribute to the advancement of the current understanding of cognitive coping in adolescents with depression, perhaps by adding some nuances that could inform tailored interventions, such as the following: (1) Severe cases of depression in adolescents that require hospitalisation share a similar pattern of cognitive coping strategies with other clinical depression populations. (2) A therapy programme aiming to increase the use of Refocusing on Planning and Positive Reappraisal, while decreasing the employment of Catastrophising, could represent an optimal solution. (3) An enhanced psychotherapeutic intervention should be developed for adolescents with depression and BPD traits, with supplementary approaches being necessary to decrease the maladaptive ways of coping (i.e., the Rumination, Catastrophising, and Blaming Others scores). (4) When depression is comorbid with either BPD traits or ASD, the therapeutic plan should also aim to decrease the use of Blaming Others as a coping strategy.

Our study faces several limitations. An important limitation of trying to describe the role of different comorbidities of depression in the cognitive coping style is represented by the absence of control groups composed of patients with these disorders but without depression (e.g., ASD, anxiety disorders, etc.). Another limitation comes from the absence in this study of a measurement to quantify depressive symptomatology, in order to improve the analysis of the relationship between the use of emotional regulation strategies and the severity of the depressive disorder. An additional limitation derives from using only self-administered measurements for emotion regulation strategies, since the obtained results are potentially biased by the comprehension of one’s own emotions. 

## 5. Conclusions

In the current study, we examined the profile of cognitive strategies used by adolescents with depression who were hospitalised in a psychiatric unit. As in previous studies, it was possible to identify a profile consisting of an increased use of two maladaptive coping strategies (e.g., Catastrophising) and the low employment of adaptive ones (i.e., Refocusing on Planning and Positive Reappraisal). Moreover, the association of depression with BPD traits led to even more frequent reports of maladaptive coping strategies (Rumination, Catastrophising, and Blaming Others). Considering that emotion regulation difficulties can be associated with psychopathology, social dysfunction, poor mental health, and wellbeing, their early identification could contribute to the development of specific, individualised preventive and intervention programmes. In addition, there is a need for further longitudinal studies to adequately measure the outcomes of these interventions.

## Figures and Tables

**Table 1:** Descriptive statistics and Cronbach’s alpha coefficients of CERQ data.

	Mean	SD	Median	Skewness	Kurtosis	Cronbach’s Alpha
MDD group (N = 112)						
Self-Blame Acceptance Rumination Positive Refocusing Refocusing on Planning Positive Reappraisal Putting into Perspective Catastrophising Blaming Others	13.52 13.63 13.71 9.88 12.84 11.21 12.11 10.51 9.15	4.78 3.38 3.86 4.85 4.26 4.67 4.06 4.25 4.08	14.00 14.00 14.00 9.00 13.00 10.00 12.00 10.00 8.00	−0.232 −0.121 −0.298 0.665 0.042 0.229 0.091 0.407 0.877	−1.128 −0.649 −0.513 −0.537 −0.963 −1.010 −0.655 −0.792 0.014	0.858 0.485 0.690 0.860 0.786 0.818 0.720 0.755 0.776
Control group (N = 78)						
Self-Blame Acceptance Rumination Positive Refocusing Refocusing on Planning Positive Reappraisal Putting into Perspective Catastrophising Blaming Others	11.54 13.72 12.97 11.01 14.99 13.97 12.79 8.40 8.91	3.32 3.71 4.19 3.54 3.46 4.09 4.39 3.33 3.19	11.00 14.00 12.00 10.00 15.00 14.00 13.00 8.00 8.50	0.473 −0.335 −0.003 0.390 −0.233 −0.238 0.064 0.707 0.718	−0.116 −0.379 −0.875 −1.033 −1.184 −0.753 −1.173 −0.151 1.60	0.736 0.746 0.803 0.578 0.740 0.819 0.793 0.752 0.776

SD = Standard deviation.

**Table 2:** Demographic data and differences in cognitive emotion regulation strategies between the MDD group and the control group.

	MDD (*n* = 112) (mean ± SD/mdn)	Control (*n* = 78) (mean ± SD/mdn)	Statistics	*p*	Effect Size
Age	14.91 ± 1.55	15.28 ± 1.89	U = 3739.0	0.08	η^2^ = 0.01
Sex					
Male Female	22 (20%) 90 (80%)	24 (30%) 54 (70%)	χ^2^ = 3.10	0.07	
Comorbidities					
Autism Spectrum Disorder BPD traits Anxiety disorders (W/o ASD or BPD) No comorbidity	26 (23%) 31 (27%) 17 (15%) 32 (28%)				
CERQ					
Self-Blame Acceptance Rumination Positive Refocusing Refocusing on Planning Positive Reappraisal Putting into Perspective Catastrophising Blaming Others	14.00 14.00 14.00 9.00 13.00 10.00 12.00 10.00 8.00	11.00 14.00 12.00 10.00 15.00 14.00 13.00 8.00 8.50	U = 3231.5 U = 4247.5 U = 3839.5 U = 3480.5 U = 3079.0 U = 2879.0 U = 4014.5 U = 3120.0 U = 4234.5	0.002 ** 0.746 0.198 0.017 * 0.001 ** 0.000 ** 0.342 0.001 ** 0.719	η^2^ = 0.05 η^2^ = 0.00 η^2^ = 0.01 η^2^ = 0.03 η^2^ = 0.06 η^2^ = 0.08 η^2^ = 0.01 η^2^ = 0.06 η^2^ = 0.00

* *p* < 0.05; ** *p* < 0.01.; SD = standard deviation; mdn = median.

**Table 3:** Differences in cognitive emotion regulation strategies in the MDD group, considering the comorbidities.

	No Comorb (*n* = 32)	ASD (*n* = 26)	BPD Traits (*n* = 31)	Anxiety (*n* = 17)	H(3)	
CERQ						
Self-Blame Acceptance Rumination Positive Refocusing Refocusing on Planning Positive Reappraisal Putting into Perspective Catastrophising	12.0 14.0 13.0 10.5 13.5 12.0 13.0 10.0	13.0 13.5 13.5 8.0 12.5 10.0 11.0 8.5	16.0 14.0 16.0 9.0 11.0 9.0 11.0 12.0	13.0 14.9 12.0 9.0 13.0 11.0 12.0 8.0	4.054 0.677 8.012 * 2.560 1.314 0.791 2.196 8.625 *	nd nd MDD + BPD > MDD + ASD/anxiety/no comorb nd nd nd nd MDD + BPD > MDD + anxiety
Blaming Others	8.0	8.5	10.0	6.0	13.878 **	MDD + BPD > MDD anxiety/no comorb; MDD + ASD > MDD anxiety

* *p* < 0.05; ** *p* < 0.01; nd = no significant difference.

## Data Availability

Data are contained within the article.
